# Potential of garnet sand as an unconventional resource of the critical high-technology metals scandium and rare earth elements

**DOI:** 10.1038/s41598-021-84614-x

**Published:** 2021-03-05

**Authors:** Franziska Klimpel, Michael Bau, Torsten Graupner

**Affiliations:** 1 Department of Physics and Earth Sciences, Jacobs University Bremen, Campus Ring 1, 28759 Bremen, Germany; 2 Bundesanstalt für Geowissenschaften und Rohstoffe, Stilleweg 2, 30655 Hannover, Germany

**Keywords:** Geochemistry, Mineralogy

## Abstract

Scandium is a critical raw material that is essential for the EU economy because of its potential application in enabling technologies such as fuel cells and lightweight materials. As there is currently no secure supply of Sc, several projects worldwide evaluate potential Sc sources. While elsewhere in Europe emphasis is placed upon secondary resources such as red mud, we investigated the potential of industrial garnet sand and its waste products. Since Sc readily substitutes for Mg and Fe in the crystal lattice of garnet, the garnet minerals almandine and pyrope, in particular, may show high Sc concentrations. Garnet sand, after being used as an abrasive in the cutting and sandblasting industry, is recycled several times before it is finally considered waste which eventually must be disposed of. Extraction of Sc (and rare earth elements, REE) from such garnet sand may generate added value and thereby reduce disposal cost. The studied garnet sands from different mines in Australia, India and the U.S., and industrial garnet sands commercially available in Germany from different suppliers show average Sc concentrations of 93.7 mg/kg and 90.7 mg/kg, respectively, i.e. similar to red mud. Our data also show that “fresh” and recycled garnet sands yield similar Sc concentrations. Within the framework of a minimum-waste approach, it may be feasible to utilize the industrial waste-product “garnet sand” as an unconventional source of Sc and REE, that reduces disposal cost.

## Introduction

In 2017, the metal scandium (Sc) was added to the list of critical raw materials (CRM) compiled by the European Union (EU)^1^ and forms part of the revised fourth CRM list of the EU as published in 2020. The 2020 CRM list comprises 30 raw materials such as the rare earth elements (REE), cobalt and lithium, that are considered essential for the European economy, while at the same time facing the challenge of high supply risk^2,3^. Due to its high cost and unstable supply, the only commercial application of Sc is currently its use in solid oxide fuel cells (SOFCs), where it allows to lower operation temperatures, which prolongs the life time of the SOFCs and reduces the material costs^4^. Moreover, Al-Sc alloys are already used in the aerospace industry and for high-end sports equipment such as baseball bats, bicycles, golf clubs and fishing rods^5^. Such Al-Sc alloys have very high potential for the automotive and aviation industries, because they have a low density while maintaining a high tensile strength^6–8^; their use, therefore, has the potential to significantly reduce the weight of an aircraft or automobile, leading to lower fuel consumption, lower emissions and lower overall costs. Despite these advantages, Al-Sc alloys are not widely used in industry yet, as there is still no secure long-term source for Sc, which results in high prices and unreliable supply on the world-market. Currently, the worldwide production of Sc is estimated to be at 15 to 20 t per year and the metal is mostly produced as a by-product^9^ as Sc only very rarely concentrates in ore-forming minerals.

While the International Union of Pure and Applied Chemistry classifies Sc as a REE^10^, its concentration in most REE deposits is insignificant due to its considerably different geochemical behavior. Scandium has an ionic radius of 75 pm in six-fold coordination and is therefore significantly smaller than the smallest REE (Lu: 86 pm)^11^, resulting in profound differences with regard to mineral-melt partitioning, for example. Moreover, REE often concentrate in a range of different discrete REE minerals, while there are only twelve terrestrial Sc minerals known yet, all of which are rare^12^.

In igneous systems, Sc is a compatible element in mafic minerals, as its ionic radius is similar to that of Mg^2+^ (72 pm) and Fe^2+^ (78 pm). Therefore, it partitions easily into major rock-forming minerals and, in particular, into clinopyroxenes^13^. During magmatic fractionation and differentiation Sc concentrations in the residual melt decrease^14^, which is in marked contrast to the REE which are incompatible elements and the concentrations of which increase in the magma during differentiation. Clinopyroxene is the most important host mineral of Sc in the majority of the deposits where Sc is currently mined as a byproduct. The largest part of the produced Sc originates from mine tailings at the giant REE deposit at Bayan Obo, China, which yield average Sc concentrations of 200 ppm. But, in contrast to the REE which reside mainly in the minerals bastnäsite ((Ce,La)CO_3_F), monazite ((Ce,La,Nd,Th)PO_4_) and xenotime (YPO_4_), Sc is hosted in the mineral aegirine ((Na, Ca)(Fe^3+^,Fe^2+^)[Si_2_O_6_]), which belongs to the clinopyroxene group^15^. An example of a Sc deposit with clinopyroxenes as major host mineral is the Zhovti Vody deposit, Ukraine^16^.

Due to the increasing economic interest in Sc, there are currently several projects worldwide that aim at developing a reliable long-term resource for Sc. Nickel- and cobalt-rich laterites developed above ultramafic to mafic bedrock in Australia appear to have the largest potential as a primary resource^17^. For some of these deposits, including the Nyngan deposit in New South Wales, mining licenses have been issued and the deposits are currently under development. The Nyngan deposit has a total Sc resource of 16.9 Mt at a 100 ppm cut-off grade, with average Sc concentrations of 235 ppm^18^. At Nyngan, Sc is usually bound to Fe oxides in the laterite^19,20^. Other laterites enriched in Sc have been found in Indonesia, Cuba and the Dominican Republic^21,22^.

In the EU member states, the focus of Sc-related research activities is on secondary rather than primary resources, in particular on industrial waste products such as red mud^23^. Red mud, a by-product of bauxite mining, shows variable Sc concentrations, ranging from 54 mg/kg in Australia to about 158 mg/kg in China^24^; Borra et al. (2015) conducted leaching experiments on bauxite residues from Greece, that show an average Sc concentration of 121 mg/kg^25^. In general, the use of secondary resources, especially of (industrial) waste products, is of increasing importance as a zero-waste strategy (as challenging as it may be) is considered an integral part of moving towards a circular economy. The concept of a circular economy has recently gained more and more attention, as the focus of metal production has moved towards a more sustainable use of resources^26^. The European Green Deal 2020 also emphasizes its importance by including “A new circular economy action plan” as a key component that builds on the circular economy actions implemented in 2015^27^.

A potential secondary source for Sc that has not been evaluated yet, may be industrial garnet sand. Based on theoretical considerations, the Fe- and Mg-rich garnet minerals almandine (Fe_3_Al_2_(SiO_4_)_3_) and pyrope (Mg_3_Al_2_(SiO_4_)_3_) may have elevated Sc concentrations, as Sc can be readily incorporated into the crystal lattice of these minerals (X^2+^_3_Y^3+^_2_(SiO_4_)_3_) and can in the eight-fold coordinated X^2+^ position substitute Fe^2+^ and Mg^2+^, respectively^28^. Scandium has been observed as a characteristic trace constituent of Mg- and Fe-rich garnets from schists (and gneisses) and to a lesser extent in garnets from pegmatites^29^. The Sc concentrations in the garnets present in the Sc rich granitic pegmatites at Tørdal in southern Norway, for example, have been found to be representative of the bulk Sc content of the pegmatites^30^.

This contribution reports on results of a pilot study to evaluate the potential of garnet sands as an unconventional resource of Sc and of rare earths and yttrium (REY). Commercially, garnet is commonly used as garnet sand for sand blasting (50%), but also for water jet cutting and water filtration^31^. This is promoted by several advantageous characteristics of garnet sands such as high hardness, high physical as well as chemical resistance, low to negligible quartz content, and high recyclability (3 to 10 times), compared to other abrasives^32,33^. In this pilot study, we investigated garnet sand samples from several mines (GS) around the world and different commercial industrial garnet sands (IGS) readily available in Germany. We compared the different garnet sands with garnet of hydrothermal origin (HG) to consider the importance of the type of garnet endmember as well as the formation processes for the Sc concentration. We determined concentrations of Sc and REY in these samples and compare them with other currently discussed secondary Sc sources to evaluate the potential of garnet sands as an unconventional source.

## Results

### Major and minor elements

The detailed results of the major and minor element analyses can be found in the supplementary Table S1, while an overview of the average concentrations is given in Table 1. The GS samples from the different mines show Al concentrations between 7.50 and 11.64 wt.%. Iron concentrations are between 21.01 and 27.44 wt.% (excluding the sample from Folkston Ore, which has 9.10 wt.%) and Mg ranges from 0.61 to 5.02 wt.%. Calcium concentrations range from 0.78 to 3.38 wt.% and Mn is between 0.41 and 9.53 wt.%.

**Table 1:** Average major element concentrations (wt.%) and REY and Sc concentrations (mg/kg) of GS, IGS and HG.

	Unit	Garnet sands from several mines (GS)	Industrial garnet sands (IGS)	Hydrothermal garnet (HG)
Al	(wt.%)	10.1	8.84	0.318
Ca	(wt.%)	1.65	1.06	19.4
Fe	(wt.%)	21.6	21.7	19.6
Mg	(wt.%)	2.98	1.94	< LOQ
Mn	(wt.%)	2.28	0.731	0.108
Sc	(mg/kg)	93.6	90.8	0.249
Y	(mg/kg)	922	221	18.8
La	(mg/kg)	1654	43.8	4.47
Ce	(mg/kg)	3498	100	12.1
Pr	(mg/kg)	407	10.3	1.31
Nd	(mg/kg)	1521	37.5	4.71
Sm	(mg/kg)	163	12.1	0.935
Eu	(mg/kg)	9.45	0.599	0.524
Gd	(mg/kg)	121	22.8	1.49
Tb	(mg/kg)	17.6	4.89	0.228
Dy	(mg/kg)	102	37.6	1.61
Ho	(mg/kg)	20.2	8.50	0.390
Er	(mg/kg)	61.8	26.8	1.37
Tm	(mg/kg)	9.90	4.04	0.200
Yb	(mg/kg)	73.4	27.3	1.48
Lu	(mg/kg)	11.5	3.99	0.267

The concentrations of Fe, Al and Mg in the IGS samples vary only slightly between the individual samples and all (except for one sample from Supplier F) show similar Fe and Al concentrations compared to the GS samples from the different deposits. Aluminum concentrations of the IGS samples, excluding the sample from supplier F, are between 7.12 and 10.86 wt.%. Iron is between 24.34 and 29.8 wt.%, while Mg ranges from 2.53 to 4.10 wt.%. Calcium concentrations fall between 0.95 and 1.80 wt.% and Mn is in the range of 0.45 to 1.14 wt.%. Major and minor element concentrations of the IGS sample from supplier F differ significantly from the other IGS with an Al concentration of only 3.11 wt.% and Fe, Mg and Ca concentrations < 0.5 wt.%.

The HG samples BF-1-I and BF-1-II have similar major element concentrations with Ca and Fe values of 19.39 and 19.26 wt.% and of 19.72 and 19.44 wt.%, respectively. The average Al concentration in the HG samples is 0.32 wt.%.

### Scandium and rare earth elements

All Sc and REY concentrations are listed in the supplementary Table S1 and an overview of the average concentrations is given in Table 1. The Sc concentrations of the GS and IGS samples are similar with averages of 93.7 mg/kg and 90.7 mg/kg, respectively. In the GS samples the Sc concentration varies between 73.6 and 120 mg/kg, when excluding the NyCor ore sample, which has a Sc concentration of only 45.4 mg/kg. All IGS except one show concentrations between 88.1 and 114 mg/kg. The exception is the IGS from Supplier F, which shows a Sc concentration of only 29.3 mg/kg. When excluding the two lowermost samples, the average Sc concentration in the GS and IGS sample sets is 103 mg/kg. In marked contrast, the few HG samples we studied show significantly lower Sc concentrations, with an average of only 0.25 mg/kg.

**Figure 1:**
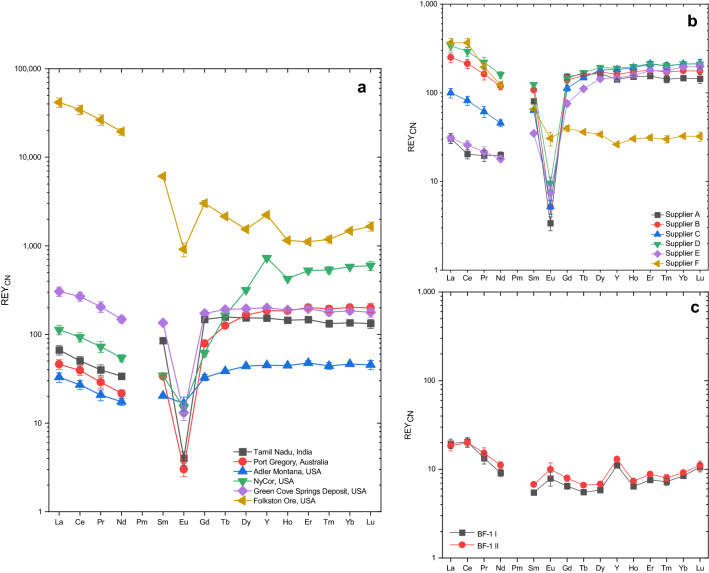
Chondrite-normalized REY patterns of garnet sand from different deposits (**a**), commercially available industrial garnet sands (**b**) and of low-temperature hydrothermal garnet from the Paleoproterozoic Beaumont Formation, South Africa (**c**). The error bars are based on 2 times the relative standard deviation (RSD) of the repeated measurement of sample Supplier C b on two different ICP-MS. Note that for some REY the error bar is smaller than the symbol size.

Most GS samples have total REY concentrations between 148 and 1,600 mg/kg, while the sample from the Folkston Ore shows a significantly higher total REY concentration of 4,800 mg/kg. The REY_CN_ patterns (Fig. 1a) are depleted in LREE_CN_ relative to HREY_CN_, except for the sample from the Green Cove Springs Deposit, which shows a very slight LREE_CN_ enrichment, and the Folkston Ore sample which shows a strong enrichment of LREE_CN_ compared to HREY_CN_. While the sample from Tamil Nadu is depleted in LREE_CN_ compared to MREE_CN_, all other samples are LREE_CN_-enriched relative to MREE_CN_. Furthermore, all samples except for the sample from the Folkston Ore are depleted in MREE_CN_ compared to HREY_CN_.

All samples are characterized by s strong negative Eu_CN_ anomaly (Eu/Eu* < 0.20, except the one from Adler Montana with 0.63) and the geochemical twins Y and Ho show no significant fractionation from the chondritic ratio (Y/Ho in garnets: 27.8–29.1, compared to the chondritic ratio of 28), except in the samples from NyCor and Folkston Ore, which both display a positive Y_CN_ anomaly, i.e. super-chondritic Y/Ho ratios of 47.3 and 53.4, respectively.

The IGS have total REY concentrations between 407 and 842 mg/kg. While the concentrations and REY_CN_ patterns (Fig. 1b) are similar for the HREY_CN_ (except for the sample from supplier F), the samples display large differences between their LREE_CN_ concentration. All samples are characterized by a general decrease from La_CN_ to Nd_CN_ for the LREE_CN_. Most samples show a relatively flat pattern for the HREY_CN_ and a large negative Eu_CN_ anomaly (Eu/Eu* < 0.12), except for the one from supplier F (Eu/Eu*: 0.55, i.e. slightly less negative). The IGS from suppliers A and E are depleted in LREE_CN_ compared to MREE_CN_, while the other samples show LREE_CN_ enrichment. Samples from suppliers B, D and F are enriched in LREE_CN_ compared to HREY_CN_, whereas the IGS from suppliers A, C and E are depleted in LREE_CN_. All samples except the one from supplier F show depletion of MREE_CN_ compared to HREY_CN_. Additionally, there is no fractionation of the geochemical twins Y and Ho for most of the IGS (Y/Ho: 25.8 to 26.3), i.e. no Y_CN_ anomaly. However, the sample from supplier F shows a slight negative Y_CN_ anomaly (Y/Ho: 23.8).

The total REY concentrations in the HG samples are 47.4 and 52.4 mg/kg, i.e. significantly lower than in the other samples. Both REY_CN_ patterns (Fig. 1c) are depleted in the MREE_CN_ and HREY_CN_ compared to the LREE_CN_ and show a relative enrichment of HREY_CN_ compared to MREE_CN_. In marked contrast to all other samples, the HG ones yield a positive Eu_CN_ anomaly (Eu/Eu* > 1.40). They also show fractionation between the geochemical twins Y and Ho, resulting in positive Y_CN_ anomalies (Y/Ho: 47.6 and 48.7, i.e. super-chondritic).

## Discussion

**Figure 2:**
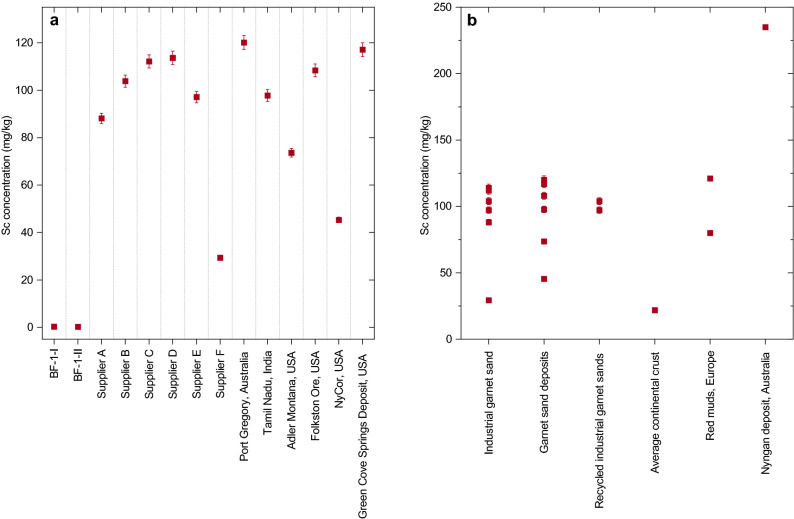
Scandium concentrations (mg/kg) of garnet from different mines, commercially available industrial garnet sands and hydrothermal garnet sands from the Beaumont Formation, South Africa (**a**). Scandium concentration in garnet sand from this study compared to average Sc concentrations in the continental crust^34^, the Nyngan Sc deposit, Australia^18^, and in red mud from Greece^25^ and Hungary^46^ (**b**). The error bars are based on 2 times the RSD of the repeated measurement of sample Supplier C b on two different ICP-MS.

The average Sc concentration of the GS and IGS samples analyzed in this study is 103 mg/kg, when the two samples with considerably lower Sc concentration are excluded. These Sc concentrations are significantly above that of average continental crust (21.9 mg/kg)^34^. In marked contrast, the HG samples are strongly depleted in Sc compared to the continental crust. The garnet sand from the NyCor deposit and that from supplier F show much lower Sc concentrations compared to those from the rest of the GS and IGS sample set (Fig. 2).

The chondritic Y/Ho ratios of most of the GS and IGS suggest that the garnets formed during metamorphism from mafic to low-silica felsic igneous or from clastic sedimentary precursor rocks as fractionated Y/Ho ratios are confined to highly evolved (SiO_2_ >  > 70%) igneous or aqueous systems^35^. In contrast, the GS samples from NYCor and Folkston ore show super-chondritic Y/Ho ratios, rather indicating highly evolved igneous precursor rocks or the involvement of larger amounts of aqueous fluid. This may also explain the difference in Sc concentration of NYCor garnets compared to the other GS. The pronounced Eu anomaly as well as the relatively flat REY_CN_ pattern from Sm and Lu indicate a garnet formation at elevated pressure^36^.

Another parameter that affects the Sc concentrations of a garnet sand is the major cation composition, i.e. the type of garnet endmember a sand sample is composed of, since the affinity of Sc for incorporation into the crystal lattice depends on a garnet’s major cations. For metamorphogenic garnets, the Sc concentration in the precursor rock is likewise important. Industrial garnet sands contain mostly pyrope-type (Mg_3_Al_2_(SiO_4_)_3_) and almandine-type (Fe^2+^_3_Al_2_(SiO_4_)_3_) garnets^37^. Since Sc preferentially substitutes Mg^2+^ and Fe^2+^ in the crystal lattice, these are also the two garnet minerals most enriched in Sc^29^. The high Fe and Al concentrations in most of the GS and IGS samples suggest that they consist mainly of almandine-type garnets.

**Figure 3:**
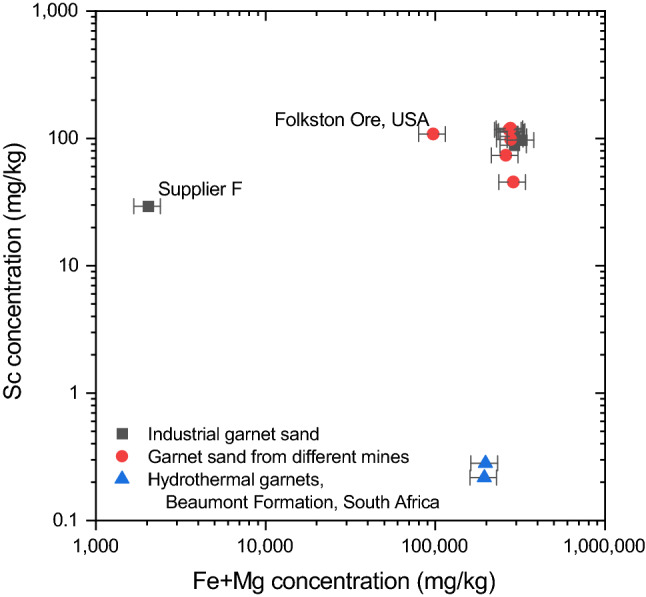
Graph of Fe + Mg concentration vs. Sc concentration (all mg/kg) for all garnet sands and hydrothermal garnets studied. The substantial variation of Sc concentrations at rather similar Fe + Mg concentrations suggests that in addition to the preferential incorporation of Sc as a substitute for Fe^2+^ and Mg^2+^ in the crystal lattice of garnets (i.e. the garnet type), the initial Sc concentrations in the parent rock also has a strong impact on the Sc concentration in garnet. The Sc error bars are based on 2 times the RSD of the repeated measurement of sample Supplier C b on two different ICP-MS. Note that for most samples the error bar is smaller than the symbol size. The Fe + Mg error bars are based on 2 times the RSD of the repeated measurement of the BHVO-2.

The low Fe and Mg concentrations, therefore, explain the considerably lower Sc concentration in the IGS sample from supplier F (Table S1 or Fig. 3). However, the strong variation in Sc concentrations throughout the complete data set, at rather similar Fe and Mg concentrations, and the characteristics of the Folkston Ore sample with its rather high Sc concentration at lower Fe and Mg concentrations (Fig. 3), suggest that the Sc concentrations in the precursor materials of the metamorphogenic garnets are also of importance.

The hydrothermal garnets from the Beaumont Formation in South Africa show very low Sc concentrations, although the garnet-precipitating fluid had interacted with Sc-rich (34.2 mg/kg) mafic igneous rocks of the Ongeluk Large Igneous Province^38–40^. The andradite formed after the hydrothermal fluid leached Ca from clinopyroxene and plagioclase^38^. The hydrothermal origin of these garnets is also evident in their REY_CN_ patterns. The positive Eu_CN_ anomalies found in both samples indicate that the fluid had experienced temperatures > 200 °C and reducing conditions^41^ prior to garnet formation at 160 to 185 °C, as suggested by fluid inclusion data^38^. The super-chondritic Y/Ho ratios also corroborate a hydrothermal rather than a metamorphic origin^35^. In contrast, the andradite present in the saprock-saprolite horizon at the Syverston-Flemington laterite, Australia, shows a high Sc concentration of ca. 250 ppm^42^, indicating that andradite is capable of incorporating Sc at significant concentrations provided the fluid is rich in Sc. At Bayan Obo, China, the mineral aegirine which hosts the largest fraction of Sc, is also supposed to be of hydrothermal origin^43^. The large range of Sc concentrations reported in these studies and in our Beaumont garnets suggest that Sc mobilization depends on fluid composition and parent rock. However, the exact processes controlling Sc mobilization during hydrothermal water–rock interaction are still poorly understood. As the hydrated ion of Sc is classified as a hard-acid after Pearson^44^, hard ligands such as hydroxide, fluoride, sulfate and phosphate may be important ligands that form stable Sc complexes in aqueous media. Unfortunately, stability constant data are only available yet for low-temperature environments. At 25 °C and 1 bar pressure, Sc will predominantly form hydroxide and fluoride complexes, depending on pH and the fluoride activity in the system^45^. The different Sc concentrations in the andradites from the Beaumont Formation and from the Syverston-Flemington Laterite emphasize the need for more experimental thermodynamic data on Sc speciation and possible Sc-complexing ligands at different temperature, pressure and ionic strength to better understand its behavior during hydrothermal processes.

Comparing GS that has been mined from the important deposits in Tamil Nadu, India, and Port Gregory, Australia, to the respective IGS product that is eventually sold to customers (Table 2), the very similar Sc concentrations as well as major and trace element compositions indicate that there is no change in chemical composition during the processing and crushing (i.e. no dilution or enrichment in garnet) (Supplementary Table S1). Furthermore, the two recycled IGS (supplier B and supplier E; Table 2) show a similar chemical composition and Sc concentration compared to the other IGS, demonstrating that the chemical composition remains unchanged during both, the industrial use and the recycling process of IGS. During this recycling process the fraction of the IGS that no longer meets the required industry standards, is separated and needs to be disposed of. The results reported here, therefore, suggest that this IGS *waste* product has a chemical composition and Sc concentration very similar to those of the GS and IGS.

As the mining and processing infrastructure for large-scale production of IGS and the supply chain already exist, there is no need to mine garnet specifically for the purpose of Sc recovery. The similar Sc concentrations of GS, IGS and *recycled* IGS indicate that the IGS waste separated during the recycling process can be considered as a potential secondary Sc source *after* its intended use in the blasting and abrasive industry. While this does not result in “zero-waste”, of course, it is yet an additional step towards this ultimate goal.

The garnet sands investigated in this study have lower average Sc concentrations than the laterites considered for mining in Australia, where the Nyngan deposit, for example, has an average concentration of 235 mg/kg^18^ (Fig. 2b). But, in contrast to the laterites, which are considered a primary resource, IGS and in particular the component in recycled IGS that has to be disposed of as a waste product, is a potential secondary source of Sc and should thus be compared to other potential secondary sources.

In the EU member states, the focus of the research activities on Sc is placed upon red mud, the waste product from alumina production from bauxite via the Bayer process. Red muds can vary in Sc concentration^24^. While for a red mud from Hungary, for example, an average Sc concentration of 80 mg/kg has been reported^46^, a red mud from Greece has higher Sc concentration with an average of 121 mg/kg^25^. In general, the Sc concentration of the industrial garnet sands considered in our study fall well-within the range of Sc concentrations found in red muds in Europe (Fig. 2b). Advantages of using red mud as a secondary resource compared to IGS is that it is available in much larger quantity and that there are large old tailings from former production. While it is estimated that 150 million tonnes of red mud are produced annually on a global scale^47^, and that a total of around 2.7 billion tonnes of red mud are theoretically available from mine tailings^48^, there are only 1.2 million tonnes of garnet sand produced worldwide^49^. On the other hand, IGS is used in various industries worldwide, which would allow for a more decentralized production of Sc. Furthermore, there already exist several methods to extract Sc from red mud^50^, some of which also allow for the simultaneous extraction of other critical metals with elevated concentrations such as REY^51^, adding economic value to the extraction process. It is currently unclear whether these methods can also be applied to extract Sc from garnet minerals.

The current price for Sc oxide (99.99%) is at 949.5 USD/kg Sc (Sc metal, 99.99%: 3140 USD/kg; September 2020)^52^. Our Sc data reveal that 1 t of industrial garnet sand contains about 100 g Sc, suggesting that 1 t of industrial garnet sand represent a Sc value of 132 USD (Sc metal: 314 USD). This also assumes that 100% of the Sc present in the garnet sands can be recovered. While this is currently under investigation, 100% extraction/recovery of a target metal during metallurgical processes is usually only rarely achieved. Therefore, even at very optimistic price assumptions for Sc, mining of garnet sand specifically for the purpose of Sc production is economically not feasible. However, the use of waste products as a secondary resource is gaining more importance and societal acceptance as the EU, for example, wants to move towards a circular economy^27^. Therefore, industrial waste products such as garnet sand waste from the cutting and sandblasting industries may be considered as an unconventional secondary Sc source in Europe, in compliance with the EU’s “Green Deal”.

As REY are also considered critical raw materials by the EU^1^, co-extraction of REY and Sc may be economically more feasible than Sc extraction alone. The total REY content of the industrial garnet sand varies between 407 and 841 mg/kg. Considering the metal oxide prices of the HREY (Dy to Lu, Y) in September 2020^52^, the HREY contained in 1 t of IGS represented a value of 16.5 USD, with Dy adding most of the value. In September 2020, the price of 1 kg of Dy oxide (99.5%) was at 256 USD^52^, implying that the Dy in 1 t of garnet sand with an average Dy concentration of 37.6 mg/kg had a value of about 11 USD. This example and the total value of the HREY indicate that the REY present in IGS do not improve the economic feasibility of critical metal extraction from IGS waste.

## Conclusion

In this pilot study we investigated GS from different mines, IGS commercially available in Germany, and garnet samples of low-temperature hydrothermal origin. Most of the GS and the IGS are significantly enriched in Sc compared to the continental crust, while the hydrothermal garnet we studied shows considerably lower concentrations. Our results suggest that not only the mineralogical composition, but also the parent rocks of the metamorphogenic garnets and their formation process(es) exert a major control on their chemical composition and, hence, their Sc content. The GS and IGS show similar Sc concentrations, indicating that the processed IGS could be used for Sc extraction. Our results also show that recycled IGS shows a chemical composition that is very close to that of fresh garnet sand, indicating that the chemical composition of the IGS is not changed by its intended use in industry. The Sc concentrations in GS and IGS are similar to those in red mud which is another secondary source considered for Sc recovery in Europe. However, extraction of Sc from GS and IGS is currently not economically feasible, as the value of the Sc presently is too low. At best, Sc extraction from IGS waste may reduce waste disposal cost and contribute towards a minimum-waste approach.

## Materials and methods

### Samples

This pilot study includes (1) samples from major garnet sand deposits in three different countries, (2) samples of industrial garnet sands commercially available from different suppliers in Germany, and (3) two hydrothermal garnet samples.

The samples of the garnet sand deposits include samples from the Tamil Nadu and the Port Gregory, deposits in India and Australia, respectively. These are the most important producers/main producing regions of industrial garnet sand worldwide. Furthermore, samples from the Adler Montana, NyCor, Folkston and Green Cove Springs deposits (all located in the USA) are included to expand the data set (Table 2).

The industrial garnet sand samples have a grain size between 120 and 300 mesh and include two recycled garnet sands, one garnet sand from India, one from South Africa and two of unknown origin (Table 2).

**Table 2:** Detailed overview over origin of samples and mesh size when applicable.

Sample type	Origin	Mesh (grain size)
Garnet sand from several mines (GS)	Tamil Nadu, India	80
	Port Gregory, Australia	80
	Adler Montana, USA	80
	Folkston Ore, USA	–
	Green Cove Springs Deposit, USA	–
	NyCor, USA	–
Industrial garnet sands (IGS)	Supplier A: unkown^a^	120
	Supplier B: recycled IGS	240
	Supplier C: India	120
	Supplier D: unkown^a^	300
	Supplier E: recycled IGS	120
	Supplier F: South Africa	120
Hydrothermal garnet (HG)	BF-1-I: Beaumont Formation, South Africa	–
	BF-1-II: Beaumont Formation, South Africa	–

^a^The supplier did not provide information on the origin of the IGS.

The two hydrothermal garnet samples are from the Early Paleoproterozoic Beaumont Formation in South Africa, which is a lateral facies-equivalent to the lower Hotazel Formation in the Transvaal Supergroup. They are comprised of andradite garnets, a calcium-iron garnet variety, and are thought to have formed hydrothermally at temperatures below 200 °C^38^.

### Sample preparation and analyses

All garnet samples (plus geological certified reference material BHVO-2, a standard that was analyzed for quality control) were dried at 105 °C overnight and 0.05 g were weighed into Teflon vessels. The samples were digested at 200 °C using a PicoTrace DAS acid pressure digestion system with a mix of suprapure HCl, HNO_3_ and HF. The acid mix was then evaporated at 200 °C to incipient dryness and the completely dissolved samples were redissolved twice in 5 ml suprapure HCl and evaporated. Finally, the samples were taken up in 0.5 M HNO_3_ for major and trace element analysis. Scandium, REY and other trace elements were analyzed by quadrupole Inductively Coupled Plasma Mass Spectrometry (ICP-MS; Perkin Elmer Nexion350x), while major elements were analyzed by Inductively Coupled Plasma Optical Emission Spectrometry (ICP-OES; SpectroCiros Vision). For ICP-MS analysis, Ru, Re, Rh and Bi, and for ICP-OES analysis Y were added as internal standards to correct for instrumental drift.

As there are several polyatomic interferences on the mass of Sc (45)^53^, the BHVO-2 reference standard and the industrial garnet sand samples were also analyzed by ICP-MS using the kinetic energy dispersion (KED) mode. In the KED mode, a He-collision cell eliminates or suppresses polyatomic interferences. As the difference between analyzing with or without KED mode was < 4.1% for all samples and < 2.03% for the BHVO-2 standard, polyatomic interferences do not affect the analyses of garnet sand samples and, therefore, all other samples were analyzed without using the KED mode (Supplementary Table S2). The method blank was below the LOQ or several orders of magnitude below the intensities measured for the garnet samples.

### Method reliability

The BHVO-2 standard was used as certified reference material and the data are compared to the reference values given by Jochum et al.^54^. The major and minor elements measured with the ICP-OES all show a relative standard deviation < 5% compared to the reference values except for Al, Fe, Mg and Na for the BHVO-2 standard digested and analyzed together with the industrial garnet sands, which have a RSD of < 15%. Scandium and other REY show RSD < 5% except for Y, Tb and Tm, which have a RSD of < 8%.

Most of the garnet samples were digested in either duplicates or triplicates and the average value is used for further evaluation. There is a variation in major and trace element concentrations of > 15% between the duplicates/triplicates of most samples. This variability is surprisingly high and has not been encountered in our laboratory in studies of other materials^39,55,56^. Nevertheless, in order to exclude analytical error, selected samples were re-measured on an Elan DRCe ICP-MS, i.e. on a different ICP-MS. These trace element values (including those of Sc and the REY) are in good agreement (RSD < 10% for most elements) with the data from the original analyses (Supplementary Table S3). Combined with the good agreement between our data for the BHVO-2 standard and its reference values, this strongly suggests that the differences between different aliquots of a garnet sand sample do not represent analytical artifacts but represent the natural inhomogeneity of the garnet sands.

### Reporting

The REY distribution is illustrated by chondrite-normalized REY patterns (subscript “CN”; chondrite data from Barrat et al.^57^ and the Eu_CN_ anomalies are quantified following Bau and Dulski as (Eu/Eu*)_CN_ = Eu_CN_/(0.67Sm_CN_ + 0.33Tb_CN_)^58^.

## Supplementary Information

Supplementary Information 1.

## References

[1] European Commission. *Communication from the Commission to the European Parliament, the Council, the European Economic and Social Committee and the Committee of the Regions on the 2017 list of Critical Raw Materials for the EU* http://eur-lex.europa.eu/legal-content/EN/TXT/PDF/?uri=CELEX:52017DC0490&from=EN (2017).

[2] European Commission. Report on critical raw materials for the EU, Report of the Ad hoc working group on defining critical raw materials. 41 (2014) Ref. Ares(2015)1819503 - 29/04/2015.

[3] European Commission (2020). Study on the Review of the List of Critical Raw Materials—Final Report. Critical Raw Materials Factsheets.

[4] Duyvesteyn WPC, Putnam GF (2014). Scandium: a review of the element, its characteristics and current and emerging commercial applications. White Paper.

[5] Emsley J (2014). Unsporting scandium. Nat. Chem..

[6] Ahmad Z (2003). The properties and application of scandium-reinforced aluminum. JOM.

[7] Naumov AV (2008). Review of the world market of rare-earth metals. Russ. J. Non-Ferr. Met..

[8] Zakharov VV (2003). Effect of scandium on the structure and properties of aluminum alloys. Met. Sci. Heat Treat..

[9] U.S. Geological Survey. Scandium. In *Mineral Commodity Summaries* (2020).

[10] Connelly NG, Damhus T, Hartshorn RM, Hutton AT (2005). Nomenclature of Inorganic Chemistry—IUPAC Recommendations 2005.

[11] Shannon RD (1976). Revised effective ionic radii and systematic studies of interatomic distances in halides and chalcogenides. Acta Crystallogr. Sect. A.

[12] Samson, I. M. & Chassé, M. Scandium. In *Encyclopedia of Geochemistry* (ed. White, W. M.) 1–4 (Springer International Publishing, 2016). 10.1007/978-3-319-39193-9.

[13] Williams-Jones AE, Vasyukova OV (2018). The economic geology of scandium, the runt of the rare earth element litter. Econ. Geol..

[14] Norman JC, Haskin LA (1968). The geochemistry of Sc: a comparison to the rare earths and Fe. Geochim. Cosmochim. Acta.

[15] Shimazaki H, Yang Z, Miyawaki R, Shigeoka M (2008). Scandium-bearing minerals in the Bayan Obo Nb-REE-Fe deposit, Inner Mongolia, China. Resour. Geol..

[16] Tarkhanov AV, Kulayev AR, Petrin AV, Kozyrkov VD (1992). The Zheltorechensk vanadium-scandium deposit. Int. Geol. Rev..

[17] Jaireth S, Hoatson DM, Miezitis Y (2014). Geological setting and resources of the major rare-earth-element deposits in Australia. Ore Geol. Rev..

[18] Rangott, M. *et al. Feasibility Study—Nyngan Scandium Project Bogan Shire, NSW, Australia NI 43-101 Technical Report* (2016).

[19] Chassé M, Griffin WLL, O’Reilly SYY, Calas G (2016). Scandium speciation in a world-class lateritic deposit. Geochem. Perspect. Lett..

[20] Qin H (2018). Chemical speciation of scandium and yttrium in laterites: new insights into the control of their partitioning behaviors. Chem. Geol..

[21] Aiglsperger T (2016). Critical metals ( REE, Sc, PGE ) in Ni laterites from Cuba and the Dominican Republic. Ore Geol. Rev..

[22] Maulana A, Sanematsu K, Sakakibara M (2016). An overview on the possibility of scandium and REE occurrence in Sulawesi, Indonesia. Indones. J. Geosci..

[23] Deady ÉA, Mouchos E, Goodenough K, Williamson BJ, Wall F (2016). A review of the potential for rare-earth element resources from European red muds: examples from Seydişehir, Turkey and Parnassus-Giona, Greece. Mineral. Mag..

[24] Ujaczki É (2018). Re-using bauxite residues: benefits beyond (critical raw) material recovery. Chem. Technol. Biotechnol..

[25] Borra CR, Pontikes Y, Binnemans K, Van Gerven T (2015). Leaching of rare earths from bauxite residue (red mud). Miner. Eng..

[26] Geissdoerfer M, Savaget P, Bocken NMP, Hultink EJ (2017). The circular economy—a new sustainability paradigm?. J. Clean. Prod..

[27] European Commission (2020). A New Circular Economy Action Plan for a Cleaner and More Competitive Europe.

[28] Chassé M, Griffin WL, Alard O, O’Reilly SY, Calas G (2018). Insights into the mantle geochemistry of scandium from a meta-analysis of garnet data. Lithos.

[29] Jaffe H (1951). The role of yttrium and other minor elements in the garnet group. Am. Mineral..

[30] Steffenssen G (2020). Unusual scandium enrichments of the Tørdal pegmatites, south Norway. Part I: garnet as Sc exploration pathfinder. Ore Geol. Rev..

[31] U.S. Geological Survey (2017). 2017 Minerals Yearbook Garnet, Industrial.

[32] Babu MK, Chetty OVK (2003). A study on recycling of abrasives in abrasive water jet machining. Wear.

[33] Olson B (2000). Garnet, Industrial.

[34] Rudnick RL, Gao S, Turekian KK, Holland HD (2014). Composition of the continental crust. Treatise on Geochemistry.

[35] Bau M (1996). Controls on the fractionation of isovalent trace elements in magmatic and aqueous systems: evidence from Y/Ho, Zr/Hf, and lanthanide tetrad effect. Contrib. Pap. Mater. Sci. Technol..

[36] Bea F, Montero P, Garuti G, Zacharini F (1997). Pressure-dependence of rare earth element distribution in amphibolite- and granulite-grade garnets. A LA-ICP-MS study. Geostand. Newsl..

[37] Elsner, H. Garnet. In *Assessment Manual—Heavy Minerals of Economic Importance* 135–160 (Bundesanstalt für Geowissenschaften und Rohstoffe, 2010).

[38] Gutzmer J (2001). Formation of jasper and andradite during low-temperature hydrothermal seafloor metamorphism, Ongeluk Formation, South Africa. Contrib. Mineral. Petrol..

[39] Schier K (2020). Chemical evolution of seawater in the Transvaal Ocean between 2426 Ma (Ongeluk Large Igneous Province) and 2413 Ma ago (Kalahari Manganese Field). Gondwana Res..

[40] Schier, K. *Personal Communication* (2020).

[41] Bau M (1991). Rare-earth element mobility during hydrothermal and metamorphic fluid-rock interaction and the significance of the oxidation state of europium. Chem. Geol..

[42] Chassé M, Griffin WL, O’Reilly SY, Calas G (2019). Australian laterites reveal mechanisms governing scandium dynamics in the critical zone. Geochim. Cosmochim. Acta.

[43] Smith MP, Henderson P (2000). Preliminary fluid inclusion constraints on fluid evolution in the Bayan Obo Fe-REE-Nb deposit, Inner Mongolia, China. Econ. Geol..

[44] Pearson RG (1963). Hard and soft acids and bases. J. Am. Chem. Soc..

[45] Wood SA, Samson IM (2006). The aqueous geochemistry of gallium, germanium, indium and scandium. Ore Geol. Rev..

[46] Ujaczki É (2017). Red mud as secondary source for critical raw materials-extraction study. J. Chem. Technol. Biotechnol..

[47] Evans K (2016). The history, challenges, and new developments in the management and use of bauxite residue. J. Sustain. Metall..

[48] Binnemans, K., Pontikes, Y., Jones, P. T., Van, T. & Blanpain, B. Recovery of rare earths from industrial waste residues : a concise review. In *3rd Int. Slag Valoris. Symp.* 191–205 (2013) 10.1016/j.jclepro.2015.02.089.

[49] U.S. Geological Survey (2020). Mineral Commodity Summaries—Garnet (Industrial).

[50] Wang W, Pranolo Y, Cheng CY (2011). Metallurgical processes for scandium recovery from various resources: a review. Hydrometallurgy.

[51] Ochsenkühn-Petropulu M, Lyberopulu T, Ochsenkühn KM, Parissakis G (1996). Recovery of lanthanides and yttrium from red mud by selective leaching. Anal. Chim. Acta.

[52] Privates Institut für Seltende Erden und Metalle e.V. Preise für Seltene Erden im September 2020 https://institut-seltene-erden.de/preise-fuer-seltene-erden-im-september-2020/ (2020).

[53] May TW (1998). A table of polyatimic interferences in ICP-MS. At. Spectrosc..

[54] Jochum KP (2015). Reference values following ISO guidelines for frequently requested rock reference materials. Geostand. Geoanal. Res..

[55] Bau M, Schmidt K, Pack A, Bendel V, Kraemer D (2018). The European shale: an improved data set for normalisation of rare earth element and yttrium concentrations in environmental and biological samples from Europe. Appl. Geochem..

[56] Schier K, Bau M, Münker C, Beukes N, Viehmann S (2018). Trace element and Nd isotope composition of shallow seawater prior to the great oxidation event: evidence from stromatolitic bioherms in the Paleoproterozoic Rooinekke and Nelani formations, South Africa. Precambrian Res..

[57] Barrat JA (2012). Geochemistry of CI chondrites: major and trace elements, and Cu and Zn Isotopes. Geochim. Cosmochim. Acta.

[58] Bau M, Dulski P (1996). Distribution of yttrium and rare-earth elements in the Penge and Kuruman iron-formations, Transvaal Supergroup, South Africa. Precambrian Res..

