# Supplementary material for: Potential of garnet sand as an unconventional resource of the critical high-technology metals scandium and rare earth elements
Source: Sci Rep. 2021 Mar 5;11:5306. doi: 10.1038/s41598-021-84614-x (PMC7970870; doi:10.1038/s41598-021-84614-x)
Supplement: Supplementary file 1 — Supplementary Information 1. [file 41598_2021_84614_MOESM1_ESM.pdf]

Potential of garnet sand as an unconventional resource of the critical high-technology metals scandium and rare earth elements

Supplementary Material

Franziska Klimpel[1*], Michael Bau[1], Torsten Graupner[2]
[1] Department of Physics and Earth Sciences, Jacobs University Bremen, Campus Ring 1 - 28759 Bremen, Germany
[2] Bundesanstalt für Geowissenschaften und Rohstoffe, Stilleweg 2, 30655 Hannover, Germany

[*]Corresponding author address: f.klimpel@jacobs-university.de

Supplementary table S1: Major, minor and trace element composition of garnet sands from different deposits (GS), industrial garnet sands (IGS) and hydrothermal garnets (HG)

Sample name	Tamil Nadu, India		Port Gregory, Australia		Adler Montana, USA		NyCor, USA	Folkston Ore, USA	Green Cove Springs Deposit, USA
number of digested samples	3		3		3		1	1	1
mg/kg	mean	%rsd	mean	%rsd	mean	%rsd			
Al	106000	0.964	101000	10.8	116000	1.13	99400	75000	111000
Ca	12800	7.41	10900	6.57	33800	1.86	7750	18100	15600
Fe	239000	1.00	245000	0.293	210000	0.42	274000	91000	234000
Mg	39300	2.82	32500	12.7	50200	2.56	13500	6130	37300
Mn	4400	2.84	8380	2	4070	1.76	14800	95300	10100
Sc	97.8	2.07	120	15.7	73.6	1.55	45.4	108	117
Y	238	1.75	291	13.5	70.1	2.00	1140	3480	312
La	15.7	30.9	10.9	36.7	7.72	48.6	26.5	9790	71.9
Ce	30.3	30.1	23.6	36.2	16.1	43.4	56.1	20700	162
Pr	3.62	25.2	2.63	35.2	1.89	32.4	6.65	2410	18.7
Nd	15.6	20.4	10	33.6	8.06	24.3	25.3	9000	68.9
Sm	13.0	4.57	5.17	21.9	3.11	8.23	5.28	930	20.7
Eu	0.235	9.31	0.176	16.8	0.977	1.30	0.87	53.7	0.763
Gd	30.4	3.39	16.3	15.3	6.73	3.87	12.7	622	35.5
Tb	5.91	2.96	4.73	13.9	1.45	2.88	6.15	80.4	7.18
Dy	39.0	2.66	41.9	12.7	11.2	1.94	80.9	391	49.5
Ho	8.20	2.49	10.4	13.5	2.52	0.963	24.1	65.2	10.7
Er	24.5	3.88	33.5	13.0	7.91	2.14	87.6	185	32.5
Tm	3.48	3.51	5.09	12.9	1.16	1.01	14.0	31.0	4.69
Yb	22.8	3.88	34.0	13.1	7.77	2.54	97.9	247	31.0
Lu	3.27	3.13	4.91	12.9	1.12	2.20	14.7	40.9	4.38

Supplementary table S1 continued

Sample name	Tamil Nadu, India	Port Gregory, Australia	Adler Montana, USA	NyCor, USA	Folkston Ore, USA	Green Cove Springs Deposit, USA
number of digested samples	3	3	3	1	1	1
mg/kg	mean	mean	mean			
Total REY content (mg/kg)	454	494	148	1600	48100	830
Y/Ho	29.1	27.9	27.8	47.3	53.4	29.1
Eu anomaly	0.0368	0.0468	0.633	0.192	0.192	0.0847
La_{CN}/Sm_{CN}	0.782	1.37	1.62	3.27	6.85	2.26
La_{CN}/Yb_{CN}	0.492	0.228	0.71	0.194	28.3	1.66
Sm_{CN}/Yb_{CN}	0.629	0.167	0.439	0.0592	4.13	0.732

Supplementary table S1 continued

Sample name	Supplier A		Supplier B		Supplier C		Supplier D		Supplier E		Supplier F	
number of digested samples	2		2		2		4		2		2	
mg/kg	mean	%rsd	mean	%rsd	mean	%rsd	mean	%rsd	mean	%rsd	mean	%rsd
Al	108000	2.6	104000	1.26	109000	0.186	107000	1.73	71200	11.6	31100	9.59
Ca	9490	7.2	9640	2.41	14800	0.122	18000	24.1	9910	6.70	2050	0.248
Fe	251000	0.176	257000	1.45	243000	0.193	251000	2.00	298000	5.48	1180	21.5
Mg	41000	2.01	36000	1.11	37900	0.423	35600	1.48	25300	14.0	854	5.84
Mn	4530	0.232	6100	1.73	6600	0.394	7900	2.54	11400	0.743	<LOQ	-
Sc	88.1	0.578	104	1.71	112	0.402	114	3.76	97.2	10.5	29.3	3.30
Y	221	0.159	252	1.82	285	0.780	294	3.55	231	9.00	40.8	1.42
La	7.17	45.5	58.8	0.857	23.5	29.0	79.8	20.5	7.29	14.4	86.3	1.88
Ce	12.2	42.8	128	0.451	49.1	26.5	176	20.0	15.4	10.6	220	1.57
Pr	1.78	39.1	14.8	0.0475	5.57	24.4	20.1	20.0	1.96	11.5	17.7	1.36
Nd	9.17	25.0	54.9	0.0981	21.1	21.5	74.3	19.5	8.36	10.9	57.0	1.29
Sm	12.3	0.168	16.4	1.41	9.70	7.55	18.9	13.6	5.33	9.69	10.0	1.29
Eu	0.198	8.43	0.302	0.81	0.304	13.6	0.558	7.39	0.44	7.90	1.79	1.73
Gd	31.3	0.616	28.7	1.23	23.2	2.23	29.9	7.08	15.6	8.80	8.19	0.622
Tb	6.08	0.597	5.84	1.63	5.56	1.47	6.33	5.09	4.18	8.15	1.35	0.535
Dy	41.7	0.105	43.7	1.80	46.0	0.430	48.9	4.20	36.5	8.32	8.58	1.08
Ho	8.57	0.106	9.73	1.35	10.9	0.652	11.2	3.90	8.91	9.51	1.71	1.21
Er	25.7	0.506	30.1	1.81	35.1	0.572	35.4	3.46	29.5	9.24	5.16	1.60
Tm	3.73	0.0448	4.47	2.48	5.26	0.110	5.31	3.11	4.67	7.95	0.785	0.665
Yb	24.6	0.601	29.8	2.22	35.6	0.235	35.7	3.17	32.9	8.95	5.44	1.17
Lu	3.54	0.0765	4.32	2.87	5.25	0.199	5.18	2.90	4.87	7.96	0.794	0.815

Supplementary table S1 continued

Sample name	Supplier A	Supplier B	Supplier C	Supplier D	Supplier E	Supplier F
number of digested samples	2	2	2	4	2	2
mg/kg	mean	mean	mean	mean	mean	mean
Total REY content (mg/kg)	409	682	561	842	407	465
Y/Ho	25.8	25.9	26.3	26.3	25.9	23.8
Eu anomaly	0.0315	0.0419	0.0567	0.0687	0.125	0.548
La_{CN}/Sm_{CN}	0.380	2.33	1.58	2.75	0.89	5.61
La_{CN}/Yb_{CN}	0.208	1.41	0.471	1.60	0.158	11.4
Sm_{CN}/Yb_{CN}	0.548	0.604	0.299	0.582	0.178	2.02

Supplementary table S1 continued

Sample name	BF-1-I	BF-1-II	BHVO-2	
number of digested samples	1	1	3	
mg/kg			mean	%rsd
Al	3240	3120	71500	11.2
Ca	194000	193000	79900	6.21
Fe	197000	194000	85200	8.03
Mg	<LOQ	<LOQ	42200	10.7
Mn	1080	<LOQ	1320	8.72
Sc	0.282	0.216	31.7	2.21
Y	17.3	20.3	23.9	2.68
La	4.60	4.33	14.6	2.25
Ce	12.2	12.0	36.9	2.06
Pr	1.22	1.39	5.14	2.40
Nd	4.24	5.18	23.4	1.42
Sm	0.839	1.03	5.96	2.28
Eu	0.461	0.587	2.01	3.71
Gd	1.33	1.64	6.17	4.15
Tb	0.207	0.248	0.883	3.90
Dy	1.48	1.74	5.21	3.51
Ho	0.363	0.417	0.939	3.93
Er	1.26	1.47	2.48	3.32
Tm	0.19	0.21	0.314	5.48
Yb	1.42	1.54	1.92	4.00
Lu	0.261	0.273	0.263	9.76
Total REY content (mg/kg)	47.4	52.4		
Y/Ho	47.6	48.7		
Eu anomaly	1.43	1.49		
La_{CN}/Sm_{CN}	3.57	2.73		
La_{CN}/Yb_{CN}	2.32	2.01		
Sm_{CN}/Yb_{CN}	0.65	0.736		

Supplementary table S2: Comparison of ICP-MS measurements of Sc with and without using KED mode

Sample name	Sc (ppm) without KED	Sc (ppm) with KED	relative standard deviation (%)
BHVO-2	31.3	32.4	2.44
Supplier A a	89.7	91.6	1.48
Supplier B a	104	107	2.01
Supplier C a	128	129	0.55
Supplier D a	107	109	1.31
Supplier E a	112	117	3.09
Supplier F a	31.4	33.1	3.73

Supplementary table S3: Comparison of ICP-MS measurements with a Perkin Elmer Nexion350x and an Elan DRCe

Sample name	Green Cove Spring Deposit			Port Gregory, Australia, a			Supplier C b			Supplier E b		
ICP-MS mg/kg	Perkin Elmer	Elan	%rsd	Perkin Elmer	Elan	%rsd	Perkin Elmer	Elan	%rsd	Perkin Elmer	Elan	%rsd
Sc	117	103	9.00	96.1	89.1	5.35	113	115	1.24	115	107	5.1
Y	312	303	2.07	237	249	3.49	283	285	0.498	232	252	5.84
La	71.9	61.1	11.5	6.09	6.64	6.11	16.7	15.3	6.19	7.44	8.34	8.07
Ce	162	138	11.3	13.4	14.4	5.09	36.0	33.1	5.94	14.8	17.1	10.2
Pr	18.7	16.4	9.27	1.53	1.62	4.04	4.21	3.81	7.05	1.96	2.18	7.52
Nd	68.9	61.1	8.49	5.98	6.36	4.35	16.6	15.6	4.39	8.29	9.27	7.89
Sm	20.7	20.6	0.342	3.81	4.23	7.39	8.97	8.84	1.03	5.42	5.85	5.40
Eu	0.763	0.756	0.652	0.147	0.156	4.2	0.262	0.231	8.89	0.343	0.405	11.7
Gd	35.5	38.9	6.46	13.1	13.8	3.68	22.7	24.0	3.94	16.5	16.9	1.69
Tb	7.18	7.89	6.66	3.86	4.41	9.41	5.47	5.58	1.41	4.20	4.52	5.19
Dy	49.5	55.2	7.70	34.7	38.9	8.07	45.8	46.4	0.92	36.3	39.6	6.15
Ho	10.7	11.8	6.91	8.46	9.40	7.44	10.8	11.0	1.30	8.87	9.76	6.76
Er	32.5	35.7	6.64	27.4	30.4	7.34	34.9	36.5	3.17	28.9	32.2	7.64
Tm	4.69	5.33	9.03	4.17	4.68	8.15	5.27	5.62	4.55	4.51	5.04	7.85
Yb	31.0	33.0	4.42	27.7	29.9	5.4	35.5	35.6	0.199	29.1	35.9	14.8
Lu	4.38	5.21	12.2	4.02	4.75	11.8	5.26	5.72	5.92	4.66	5.26	8.55
